# Charles Frick, M.D.

**Published:** 1860-05

**Authors:** 


### NECROLOGICAL NOTICES.

CHARLES FRICK, M.D.—It is our melancholy duty to announce the death of this amiable, excellent, and intelligent physician, at the early age of thirty-seven, from diphtheria, at his residence in Baltimore, on Sunday, the twenty-fifth ultimo. The disease which thus cut off, in the prime and vigor of his manhood, one of the most promising members of the profession, ran its course with great rapidity, and baffled all the skill and devoted attention of his professional friends and attendants. Tracheotomy was performed upon Dr. Frick about nine hours before dissolution, with, however, hardly any amelioration of his condition.

Dr. Frick occupied, at the time of his lamented demise, the Chair of Materia Medica in the University of Maryland, a position to which he was elevated not quite two years ago, having previously filled a similar office in the Baltimore College of Pharmacy. He was a son of the late Judge Frick, and a nephew of Dr. Frick, the author of a work on Diseases of the Eye, based principally upon the writings of Beer, and other German oculists. He himself published, several years ago, an excellent little work, the result of original observations, on urinary deposits, and a short time before his death he contributed an admirable lecture on diuretic remedies, full of practical value, to the pages of the *Maryland and Virginia Medical Journal.*

Professor Frick was a gentleman of the most genial feeling, of unusual intelligence, cultivated and agreeable manners, excellent colloquial powers, and high professional ambition. He leaves behind him a wife and an only child to lament his untimely loss; a loss which will be deeply deplored by the whole American profession. Personally, we feel as if we had lost a kind friend, the recollection of whose manly virtues and great promise overwhelms us with the deepest sadness.

